# Retinal Vasculature and Microstructure in Early Dry-Type Myopic Maculopathy

**DOI:** 10.1155/2019/7540897

**Published:** 2019-08-05

**Authors:** Jiao Sun, Jialin Wang, Yanling Wang

**Affiliations:** Department of Ophthalmology, Beijing Friendship Hospital Affiliated to Capital Medical University, Beijing, China

## Abstract

**Purpose:**

The aim of this cross-sectional study was to characterize and compare the retinal vasculature and microstructure in patients with early dry-type myopic maculopathy.

**Methods:**

Patients with a refractive error of less than −6 diopters were enrolled and classified into two groups. Group 1 comprised 82 eyes with a tessellated fundus, and group 2 comprised 56 eyes with diffuse chorioretinal atrophy (DCA). The clinical characteristics, refractive error, axial length, retinal vessel density of the superficial capillary plexus (SCP) and deep capillary plexus (DCP), macular choroidal thickness, and best-corrected visual acuity (BCVA), were compared between the groups. Logistic regression was used to determine the protective and risk factors for DCA.

**Results:**

Group 1 patients were significantly younger and had better BCVA, less myopia, and shorter axial length than group 2 patients. The vessel densities of the SCP and DCP, choroidal thickness, retinal nerve fiber layer thickness, ganglion cell complex thickness, and retinal thickness were reduced in group 2. Multiple logistic regression analysis revealed that the vessel densities of the SCP and DCP were protective factors for DCA.

**Conclusions:**

The vessel density of the SCP had the highest diagnostic value (sensitivity = 78.0% and specificity = 96.6%). When the SCP vessel density was reduced to ≤49.98%, DCA was indicated. The retinal vessel densities of the SCP and DCP and parameters of the microstructure were reduced significantly in patients with DCA. Vessel density may be a better diagnostic indicator of the development of DCA.

## 1. Introduction

Myopic maculopathy is a major cause of irreversible visual impairment and blindness worldwide, particularly in East Asia. The Beijing Eye Study [1] and Shihpai Eye Study [2] revealed that myopic maculopathy is the second most frequent cause of low vision and blindness in China, which is more prevalent than age-related macular degeneration (AMD).

Myopic maculopathy can be classified as either wet- or dry-type AMD depending on the presence or absence of choroidal neovascularization (CNV), respectively [3]. In a new grading system for pathological myopia (META-PM classification), early dry-type myopia equates to tessellated fundus (category 1 (C1)) and diffuse chorioretinal atrophy (DCA) (category 2 (C2)) [4]. The appearance of tessellated fundus in the eye may act as a marker for determining visual performance, the degree of myopia, or the risk of myopia progression [5]. DCA is a vision-threatening complication of high myopia wherein the best-corrected visual acuity (BCVA) gradually worsens with foveal involvement. Highly myopic eyes with DCA have poorer BCVA and thinner macular choroidal thickness than eyes with a tessellated fundus [3]. Furthermore, DCA is becoming more frequently recognized as a progressive lesion associated with visual impairment. A series of critical sight-threatening pathological consequences may appear due to DCA, such as lacquer cracks, CNV, patchy atrophy, and macular atrophy. Effective therapies for myopic CNV are available; however, there is no treatment available for dry-type myopic maculopathy. Eyes with tessellated fundus, lacquer cracks, diffuse atrophy, and patchy atrophy at the initial examination may exhibit CNV development [6]. Therefore, the early detection of highly susceptible eyes among those with dry-type myopic maculopathy is crucial.

Morphological changes in the vasculature and structure of the retina in patients with myopia have been observed previously. Reductions in retinal blood flow and thinning of the choroid in healthy highly myopic eyes might relate to the development of myopic chorioretinal atrophy [3, 7]. Previous studies had many limitations due to imaging modalities; therefore, they could only focus on large vessels and use invasive methods such as fluorescein angiography. However, with the recent advances in optical coherence tomography angiography (OCTA), vasculature can be measured quantitatively in a noninvasive manner and provides morphological information, which can expand our understanding of the retinal vasculature and microstructure in patients with early-stage dry myopia.

Therefore, the purpose of the current study was to characterize alterations in the retinal vasculature and microstructure in two types of early myopic maculopathy and to determine the risk factors and development of DCA.

## 2. Methods

### 2.1. Participants

This cross-sectional study was conducted in compliance with the tenets of the Declaration of Helsinki. Informed consent was obtained from all examined patients to participate in this research. Patients from the Beijing Friendship Hospital (Beijing, China) were recruited. Patients underwent comprehensive ophthalmologic examinations, including measurement of the BCVA, slit-lamp examination, fundus color photography (Kowa Nonmyd WX; Kowa Company Ltd., Japan), intraocular pressure (IOP), and axial length (AL) measurement by optical biometry (IOLMaster; Carl Zeiss Meditec, Jena, Germany).

Participants with a refractive error of less than −6 diopters (D) or an AL of >26.5 mm were recruited for this study. The exclusion criteria were any history or clinical evidence of vitreoretinal conditions or surgery: IOP of >21 mmHg, visual field defects, systemic diseases potentially affecting the eyes, opaque media, myopic maculopathy worse than C2 based on the META-PM classification (including patchy atrophy (C3) and macular atrophy (C4)), and pathological structures resulting from spectral-domain OCT, such as CNV, foveoschisis, epiretinal membrane, retinal detachment, and macular hole.

**Figure 1:**
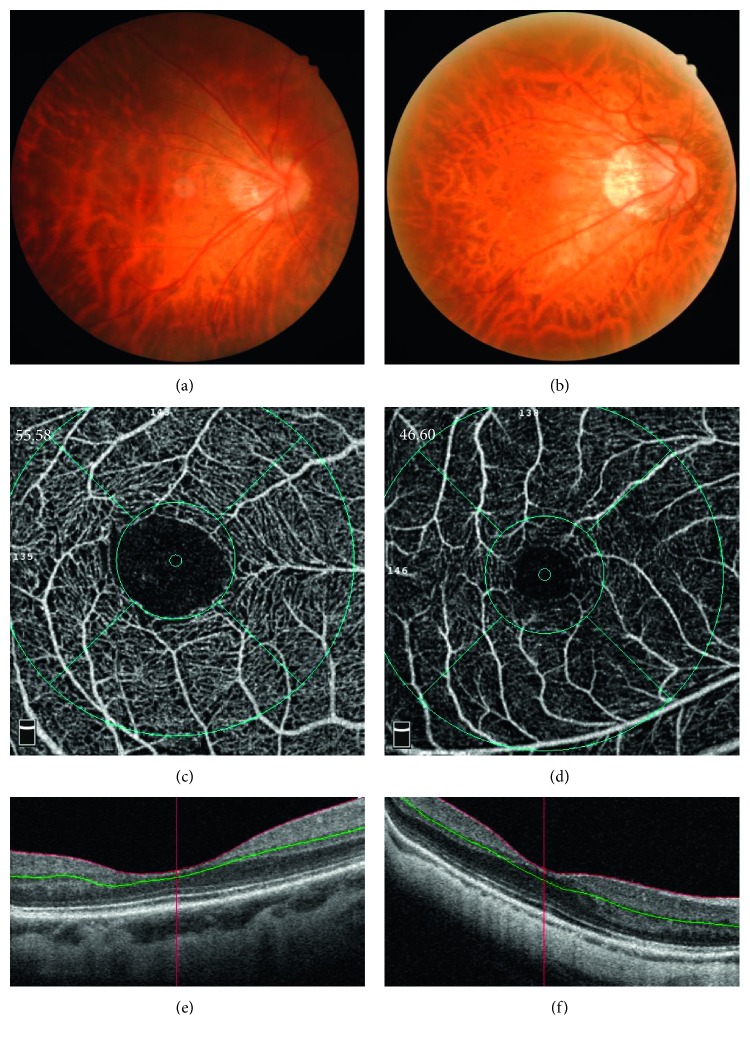
Representative images of eyes with early dry-type myopic maculopathy. A 37-year-old female patient with tessellated fundus (a), superficial vessel density of 52.05 (c), and thick choroid (e). A 40-year-old female patient with diffuse chorioretinal atrophy (b), superficial vessel density of 46.60 (d), and a thinner choroid than in the left (f).

The eyes were categorized into two groups according to the META-PM classification [4]: group 1, eyes with only tessellated fundus (C1), and group 2, eyes with only DCA (C2). We compared the clinical characteristics, microstructure, and vasculature between these two groups (Figure 1).

### 2.2. OCTA Measurements

The OCTA was performed with an Avanti RTVue XR system (Optovue Inc., Fremont, California, USA). The instrument has an A-scan rate of 70,000 scans per second. The acquisition time per volume amounted to approximately 2.9 s for each of the two raster scans. The resolution of the exported OCTA images was 304 × 304 pixels. The images were obtained with the macula protocol with an area of 3 mm × 3 mm. The automated segmentation with the preset settings for the superficial capillary plexus (SCP) and deep retinal capillary plexus (DCP) was utilized. The upper border of the superficial vascular layer was defined as 3 *μ*m below the internal limiting membrane (ILM) and the lower border as 15 *μ*m below the inner plexiform layer (IPL). The borders of DCP were defined as 15 *μ*m and 70 *μ*m below the IPL, respectively. The imaging density used in myopic eyes must be lower than that used in normal eyes because the magnification is different in myopic eyes. Therefore, Bennett's formula was used to correct the magnification of images obtained from highly myopic eyes. The retinal nerve fiber layer (RNFL) thickness, ganglion cell complex (GCC) thickness, and retinal thickness were also measured by the RTVue instrument. Two independent examiners reviewed the images.

### 2.3. OCT Measurements

All the subjects were examined with the RTVue XR Avanti OCT instrument. The peripapillary RNFL and macular GCC thicknesses were measured. The GCC scanning protocol consisted of a 7 × 7 mm area, which measured the retinal thickness from the ILM to the IPL posterior boundary.

The retinal thickness of the macular area was also measured using the RTVue XR Avanti OCT instrument. The full retinal thickness was measured from the ILM to the outer boundary of the retinal pigment epithelium (RPE). Eye movements were monitored by reading the real-time fundus images. We obtained average retinal thickness measurements in six subfield regions, according to the Early Treatment Diabetic Retinopathy Study. Two trained ophthalmologists examined and assessed the image quality independently.

In addition, raster and macular scans obtained by high-definition spectral-domain OCT were used to examine the retinal and choroidal integrity and to determine the presence/absence of myopic CNV and patchy atrophy. The choroidal thickness, defined as the distance between the RPE and the scleral interface (reflective line beneath the fovea), was manually measured by two masked observers using enhanced-depth imaging OCT images and Heidelberg Eye Explorer software (Heidelberg Engineering GmbH, Heidelberg, Germany). The final choroidal thickness was recorded as the average of the two independent measurements.

### 2.4. Statistical Analysis

Statistical analyses were performed using SPSS statistical software (version 24.0, SPSS, Inc., Chicago, Illinois, USA). Statistical significance was defined as *P* < 0.05. Data are presented as the mean ± standard deviation. The Kolmogorov–Smirnov test was used to determine normal distributions. An independent *t*-test was used to perform comparisons between the groups. Categorical variables were compared using the chi-square or Fisher's exact test. Multivariate logistic regression analyses were conducted to determine the odds ratio (OR) for factors identified as significant in the stepwise regression analysis. The following three models were used to perform regression analyses: Model 1 = crude model, Model 2 = adjusted for sex and age, and Model 3 = adjusted for choroidal thickness and AL. Furthermore, receiver operating characteristic (ROC) curves were generated, and the area under the curve (AUC) was applied to assess the properties of biometric parameters in perceiving diffuse atrophy.

## 3. Results

### 3.1. Comparison of Characteristics in Patients with Early Dry-Type Myopic Maculopathy

Group 1 comprised 82 eyes of 50 patients, and group 2 comprised 56 eyes of 40 patients. Group 1 patients were significantly younger than those in group 2 (*P*=0.034) (Table 1). The refractive error and the mean logarithm of minimal angle of resolution visual acuity were significantly smaller in group 1 than in group 2 (*P* < 0.001 for both). The AL was significantly shorter in group 1 than in group 2 (*P*=0.001). The mean macular choroidal thickness was significantly greater in group 1 than in group 2 (*P*=0.001).

### 3.2. Comparison of Vessel Density, RNFL, GCC, and Retinal Thickness in Patients with Early Dry-Type Myopic Maculopathy

**Figure 2:**
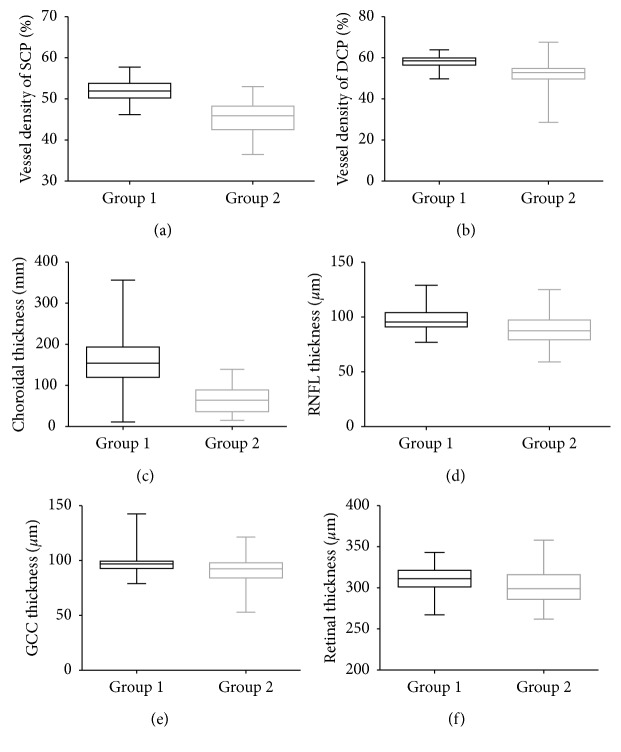
Comparison of the vessel density in the superficial retinal capillary plexus (SCP) (a), vessel density in the deep retinal capillary plexus (DCP) (b), choroidal thickness (c), retinal nerve fiber layer (RNFL) thickness (d), ganglion cell complex (GCC) thickness (e), and retinal thickness (f) in groups 1 and 2. All differences between groups 1 and 2 are significant (*P* < 0.05).

The mean vessel densities in the SCP and DCP were significantly greater in group 1 than in group 2 (*P* < 0.001 for both) (Table 2 and Figure 2). Furthermore, comparison of the two layers in each of the four different sectors indicated that the differences in the vessel densities were statistically significant (*P* < 0.001). We concluded that the RNFL thickness and retinal thickness were different between the two groups in different locations. Significant differences were found in the mean GCC thickness between groups 1 and 2 (*P* < 0.001).

### 3.3. Multiple Logistic Regression Analysis of Factors Associated with DCA

Logistic regression analysis revealed that the vessel densities in the SCP and DCP was protective factors for DCA (ORs = 0.476 and 0.613, respectively; *P* < 0.001 for both). Furthermore, the third logistic regression model indicated that the choroid thickness was a protective factor for DCA (OR = 0.975, *P*=0.025). However, age and AL were risk factors for DCA (ORs = 1.218 and 5.579, respectively; *P*=0.001 for both) (Table 3).

### 3.4. ROC Analysis of the Vessel Density and Choroidal Thickness for the Diagnosis of DCA (C2)

**Figure 3:**
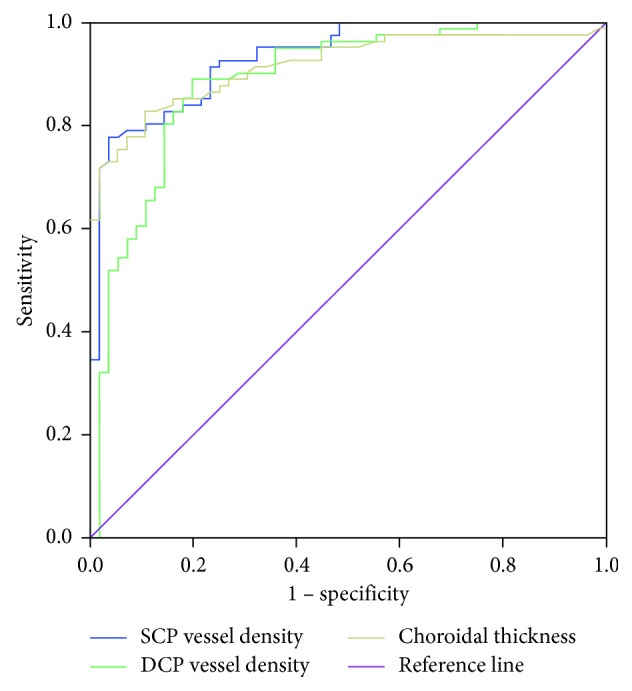
Receiver operating characteristic curves of the vessel densities in the superficial retinal capillary plexus and deep retinal capillary plexus and choroidal thickness for predicting diffuse chorioretinal atrophy. SCP = superficial retinal capillary plexus; DCP = deep retinal capillary plexus.

Using ROC analysis of ocular parameters for detecting DCA in highly myopic eyes, the largest AUC was for the SCP vessel density, followed by the AUCs for choroidal thickness and DCP vessel density (all *P* < 0.001) (Table 4). Thus, the SCP vessel density had the highest diagnostic value (sensitivity = 78.0% and specificity = 96.6%). When the SCP vessel density was reduced to ≤49.98%, DCA was indicated (Table 4 and Figure 3).

## 4. Discussion

Here, we demonstrated differences in vasculature and microstructure between the eyes with a tessellated fundus and the eyes with DCA in patients with early dry-type myopic maculopathy. Furthermore, a decrease in vessel density was a significant risk factor for the development of DCA. Owing to earlier limitations of imaging modalities, previous studies only focused on large vessels using invasive methods. Furthermore, these studies did not consider the retinal vasculature and its comparison in such early stages. To our knowledge, our analysis is the first to determine the vasculature and microstructural alterations in the retina between tessellation and DCA, which may provide a better understanding of the pathogenesis of myopia.

Groups 1 and 2 represent two different categories or stages of myopic maculopathy with regard to their clinical features and significance. Early DCA development may be a potential biomarker for more advanced pathological myopia in later life [8]. Patients with DCA were significantly older and had poorer BCVA, more severe myopia, longer AL, and thinner choroidal thickness. Previous studies also reported hese findings and further classified them into detailed subgroups [9]. Tessellation might be a relatively stable stage, and high myopia might be delayed. Tessellation alone does not typically cause decreased visual acuity, as myopic maculopathy tends to progress more quickly after it has advanced past the tessellated fundus stage [6]. Eyes with DCA had poorer BCVA than those without. There are two possible explanations for this decline. The first is the thinner choroidal thickness in eyes with DCA. Choroidal vessels provide the RPE cells and photoreceptor cells with nutrients and oxygen. Thinning of the choroid may affect the function of these cells due to ischemia, which may lead to vision loss. Second, the retinal vasculature in both the SCP and DCP, which provide nutrients to the retina, decreases significantly in DCA.

In our study, the retinal vessel density in the SCP and DCP decreased more significantly in DCA than tessellation. Previous studies using different imaging modalities have demonstrated a reduction in retinal and choroidal perfusion in patients with high myopia. With the development of OCTA, more severe myopia was demonstrated to be associated with decreased vascular density in the macula, which is consistent with our study [10]. Blood flow in the retinal arteries was found to be decreased in highly myopic eyes compared to that in emmetropic or mildly myopic eyes using laser Doppler velocimetry [11], and the diameter of the retinal arteries becomes smaller with myopic change [12]. Recent studies including our previous research demonstrated that the densities in the superficial and deep microvascular plexuses were significantly decreased in patients with nonpathological high myopia [13, 14]. However, another study concluded that varying degrees of myopia did not affect the macular vascular density in young healthy adults with tessellation [15].

To our knowledge, our study is the first to investigate the retinal vasculature of DCA alone. Earlier studies focused on the choroid in DCA or different stages of myopia. The retina has three levels of blood supply (plexuses): the radial peripapillary capillary plexus, superficial plexuses, and deep plexuses. Half of the inner retina receives blood supply from three of these vascular plexuses, whereas the choriocapillaries provide blood supply to the outer half of the retina and macula; these are derived from the posterior ciliary artery [16]. Due to elongation of the AL, the density of the choroidal vessels and choroid thickness decrease markedly in the area of diffuse atrophy; therefore, the supply from the choriocapillaries is reduced. Furthermore, the thinning and atrophy of the retinal tissue may cause reduced oxygen demand, resulting in decreased blood circulation.

Earlier studies have reported an association between glaucoma and myopia; however, the mechanism by which myopia increases the risk of glaucoma remains unclear. The macular vessel density is decreased in glaucoma and high myopia and is correlated with the extent of disease. The RNFL and GCC are both highly significant and characteristic parameters in glaucoma. Reduced peripapillary perfusion can lead to atrophy or lesion of the optic nerve head, RNFL thinning, and visual field defects. Together, these factors can increase the risk of normal-tension glaucoma (NTG) in high myopia. However, whether the reduced vascular perfusion in high myopia is a cause or result of NTG is not clear. The GCC in the macular area is thinner in patients with DCA, and this can be attributed to the vascular decline. Many studies have demonstrated that ischemia has multiple detrimental effects on retinal ganglion cells (RGCs) and their axons. The mechanisms implicated in RGC death include hypoxia-induced reactive oxygen species, excitotoxicity, nitric oxide, and inflammation. There is strong evidence that chronic retinal ischemia also results in a variety of harmful intraretinal events [17] and reduced retinal thickness in patients with DCA. Myopization correlates with axial elongation, resulting in biomechanical stretching of the retina, choroid, and sclera, which could cause a reduction in the retinal thickness [18]. Elucidating the effect of myopia on ocular circulation can lead to improved knowledge of the pathogenesis of myopia.

Using multivariate logistic regression, we found that the vessel densities in the SCP and DCP are both protective factors for DCA, even after adjusting for age, sex, AL, and choroidal thickness. Since the whole retina is perfused by both choroidal and retinal vasculature systems [19] and based on our finding that the retinal microstructure was thinner due to DCA, it is unreasonable and unilateral to only study the choroidal vasculature as in earlier studies. ROC curve analysis indicated that the AUC for the vessel density in the SCP was the highest, followed by choroidal thickness and the vessel density in the DCP, which can detect DCA in high myopia. In our study, when the vessel density was reduced to 49.98%, we considered DCA in the fundus. We speculated that the choroidal and retinal degeneration in patients with DCA occurred due to ischemia. These results might also be due to the subjective measurement using OCTA.

There are several strengths to this study. First, unlike previous studies, which only considered choroidal vasculature changes and did not categorize participants according to the features of the fundus, our analysis is the first to study the difference in vasculature and microstructure in two subgroups of early-type dry myopia. Second, we investigated the characteristics using a novel quantitative method, i.e., OCTA. Our findings can increase the clinical significance of vessel density and benefit patients in the early diagnosis and detection of DCA in those with high myopia.

There are several limitations to our study. First, the design was cross-sectional and the sample size was small, warranting long-term studies with larger sample sizes and close follow-up. Second, we assumed that all of the eyes were independent of one another; however, some eyes may have belonged to the same patients.

In conclusion, our study demonstrated a difference between tessellation and DCA and retinal vessel density, which can be measured noninvasively and provide earlier detailed information. Thus, retinal vessel density may become a novel marker for the detection of DCA and enable differentiation from highly myopic tessellation. These results are clinically valuable for predicting the progression of myopic maculopathy in patients with high myopia. Thus, identifying methods to increase retinal and choroidal perfusion in patients with high myopia, particularly dry type, is required in future studies.

## Figures and Tables

**Table 1:** Comparison of demographic and clinical data and retinal vessel density in patients with early dry-type myopic maculopathy.

	Group 1 (*n* = 82)	Group 2 (*n* = 56)	*P*
Age (years)	38.13 ± 13.03	59.79 ± 9.94	0.035
Sex (male, %)	39.02	26.83	0.862
IOP (mmHg)	15.74 ± 2.96	15.66 ± 2.55	0.907
Refractive error (D)	9.16 ± 3.01	12.71 ± 5.14	<0.001
BCVA, logMAR	0.07 ± 0.16	0.30 ± 0.28	<0.001
Axial length (mm)	26.79 ± 1.19	28.88 ± 1.81	0.001
Macular choroidal thickness (*μ*m)	162.46 ± 69.29	63.29 ± 32.54	<0.001

IOP = intraocular pressure; D = diopters; BCVA = best-corrected visual acuity; logMAR = logarithm of the minimal angle of resolution. Data are expressed as the mean ± standard deviation unless otherwise indicated.

**Table 2:** Comparison of the vessel density, retinal nerve fiber layer, ganglion cell complex, and retinal thickness in patients with early dry-type myopic maculopathy.

	Group 1 (*n* = 82)	Group 2 (*n* = 56)	*P*
Vessel density in the SCP (%)				
Mean	51.93 ± 2.71	45.24 ± 3.83	<0.001
Parafovea	54.65 ± 3.03	47.56 ± 4.30	<0.001
Temporal	54.08 ± 3.21	47.49 ± 7.35	<0.001
Superior	56.25 ± 3.01	49.47 ± 5.07	<0.001
Nasal	54.10 ± 3.66	46.49 ± 5.32	<0.001
Inferior	54.19 ± 3.92	46.40 ± 5.21	<0.001
Vessel density in the DCP (%)				
Mean	58.02 ± 2.61	51.89 ± 5.63	<0.001
Parafovea	63.75 ± 2.86	57.69 ± 6.19	<0.001
Temporal	61.81 ± 3.41	56.70 ± 6.57	<0.001
Superior	64.85 ± 7.17	59.58 ± 5.87	<0.001
Nasal	62.59 ± 3.61	57.42 ± 8.79	<0.001
Inferior	64.97 ± 3.55	57.69 ± 7.77	<0.001
RNFL thickness (*μ*m)				
Mean	98.57 ± 10.62	89.23 ± 14.36	<0.001
Superior	102.46 ± 12.22	93.65 ± 17.60	0.001
Inferior	94.70 ± 11.23	84.83 ± 15.30	<0.001
GCC thickness (*μ*m)			
Mean	96.71 ± 8.37	92.42 ± 11.35	0.006
Superior	96.81 ± 9.39	93.34 ± 11.70	0.051
Inferior	96.63 ± 7.89	91.64 ± 12.02	0.001
Retinal thickness (*μ*m)				
Parafovea	310.30 ± 15.81	300.97 ± 20.96	0.001
Temporal	302.13 ± 16.05	292.64 ± 19.34	0.001
Superior	316.21 ± 17.22	305.44 ± 25.20	0.001
Nasal	314.29 ± 18.42	306.86 ± 26.00	0.014
Inferior	309.02 ± 14.95	299.22 ± 21.17	<0.001

Data are expressed as the mean ± standard deviation unless otherwise indicated. SCP = superficial retinal capillary plexus; DCP = deep retinal capillary plexus; RNFL = retinal nerve fiber layer; GCC = ganglion cell complex.

**Table 3:** Logistic regression analysis of factors associated with diffuse chorioretinal atrophy.

		Unstandardized coefficients		*P*	OR	95% CI for *B*	
		*B*	Standard error			Lower bound	Upper bound
SCP							
Model 1	Vessel density	−0.742	0.132	<0.001	0.476	0.367	0.617
Model 2	Vessel density	−0.633	0.145	<0.001	0.531	0.400	0.706
Model 3	Vessel density	−0.571	0.200	0.004	0.565	0.382	0.835
	Age	0.154	0.059	0.009	1.167	1.040	1.309
	Sex	−2.708	1.297	0.037	0.067	0.005	0.847
	CT	−0.029	0.013	0.030	0.972	0.947	0.997
	AL	1.812	0.674	0.007	6.122	1.632	22.960
DCP							
Model 1	Vessel density	−0.489	0.086	<0.001	0.613	0.518	0.726
Model 2	Vessel density	−0.380	0.098	<0.001	0.684	0.564	0.829
Model 3	Vessel density	−0.311	0.154	0.043	0.732	0.542	0.990
	Age	0.197	0.058	0.001	1.218	1.087	1.366
	Sex	−2.167	1.061	0.041	0.115	0.014	0.916
	CT	−0.025	0.011	0.025	0.975	0.953	0.997
	AL	1.719	0.530	0.001	5.579	1.973	15.780

Model 1 = crude model; Model 2 = further adjustment for sex and age; Model 3 = further adjustment for axial length and choroidal thickness; OR = odds ratio; B = regression correlation coefficient; CI = confidence interval; SCP = superficial retinal capillary plexus; DCP = deep retinal capillary plexus; CT = choroidal thickness; AL = axial length.

**Table 4:** The area under the curve and cutoff values of vessel density and choroidal thickness for differential diagnosis of diffuse chorioretinal atrophy (C2).

	AUC	95% CI	Cut-off point	*P*	Sensitivity	Specificity
SCP vessel density	0.932	0.892–0.972	≥49.98	<0.001	0.780	0.966
DCP vessel density	0.888	0.830–0.947	≥55.15	<0.001	0.889	0.804
Choroidal thickness	0.915	0.868–0.963	≥102.50	<0.001	0.829	0.881

AUC = area under curve; CI = confidence interval; SCP = superficial retinal capillary plexus; DCP = deep retinal capillary plexus.

## Data Availability

The data used to support the findings of this study are included within the article.
